# Some Facts Regarding Reorganization in Other States

**Published:** 1905-04

**Authors:** 


### CORRESPONDENCE.

### SOME FACTS REGARDING REORGANIZATION IN OTHER STATES.

Desiring to place before the members of the Association as much information as possible bearing upon the results of re-organization in other States, your committee wrote to the officers of a number of Associations asking that they would give conditions existing in their States before and since the adoption of the constitution suggested by the American Medical Association.

Prompt replies were received in every instance and the following facts are taken from the letters of either the secretary or the president of the Associations of the States named.

In Kentucky, prior to the adoption of the new constitution, there were about 400 members in the State Association, with less than 100 members usually attending an annual meeting. At the present time, two years after the adoption of the proposed constitution, there are 1,600 paid-up members, a large increase in the attendance upon meetings and a general awakening of the profession at large.

In Michigan, prior to 1902, the State membership had never exceeded 500, last year it was 1,800 paid-up members. Formerly only eleven counties in the State had any kind of medical society, now every county in the State is organized and working in harmony with the State body.

In Tennessee, prior to the adoption of the new constitution, the membership had at no time been greater than 400, now it has passed 1,200; dues formerly collected had rarely been $700.00, last year it was over $2,000.00, while the quantity and character of scientific work done has been much improved.

In Indiana, while the proposed plan was already virtually in existence, the State being organized on the basis of the county unit, still the membership increased twenty-five per cent. the first year, and a number of unorganized counties at once formed societies and sent delegates to the State Association. No move in the State has ever given such promise of bringing all the medical men into societies for their scientific and social advancement.

In Nebraska, the membership increased the first year from 275 to about 800, and the sentiment is heartily in favor of the change.

In Louisiana the new constitution was adopted in 1903, and reports a considerable increase in membership and a corresponding interest in scientific work. The president expresses satisfaction with the plan and writes that it is working well.

Illinois reports that membership in the State Association has increased fourfold, that there are now between 4,000 and 5,000 members in the Association, or about 60 per cent. of the regular physicians in the State with a live working unit in every county. Interest in medical matters has increased correspondingly and the rank and file of the profession are more than pleased with the change.

Arkansas writes that in one year the membership of the Association went from 150 to 700, and that the plan has given general satisfaction.

Wisconsin had in 1903 in her State Association 700 members and there were ten feeble county societies in the State. The following year, after adopting the new constitution, the membership increased to 1,300, while every county in the State became organized and now have live working societies. At the meeting of 1904 there were 100 more members in attendance than at any previous meeting within the history of the Association.

In the neighboring State of Florida, the president, Dr. Pierpont, writes in the most enthusiastic manner. He states that the membership has increased from 172 to 243, but it is especially in connection with the work of the county societies that he speaks most enthusiastically. Here, he says, the results have been most gratifying.

Dr. Chase, of Fort Worth, the secretary of the Texas State Association, writes that prior to reorganization, or in April, 1903, there were 445 members, only about 10 per cent. of the regular practitioners, while the collections from all sources amounted to $1,815.00. In April, 1904, or one year later, the membership was 2,393, while the treasurer reported collections amounting to $5,986.00. Thus, you see the increase was fivefold the first year and the Association now has enrolled on its list over half of the legalized practitioners of the State. This in a State 1,000 miles square. Dr. Chase goes on to say that there has never been any five-year period in the history of the State in which there has been so great an increase in good feeling among the profession, or in which more irregular practitioners have been forced out of practice or in which so much political influence for good has been exerted.

From North Carolina, we have letters from both the president and the secretary of the Association. The president, Dr. Tayloe, says, "Our medical society, now running under the plan suggested by the American Medical Association, has met with the universal approval of 99 per cent. of the doctors of our State. It has more than doubled the membership, put twice the money in the treasury and worked a success thus far in every particular." The secretary, Dr. Way, of Waynesville, whom some of you may know, a fine fellow, says, "I beg leave to say to you most emphatically, *reorganize* and do it *now!*" He then goes on to say that the membership increased in one year from 283 to 1,065 paid members, that 90 out of 97 counties have organized, and that owing to thorough organization, they had no trouble whatever in killing before the State legislature a bill designed to recognize osteopaths.

F. W. McRAE,
*Chairman Com. on New Constitution.*

---

BOWLING GREEN, KY., March 18, 1905.

*Dr. Louis H. Jones, Secretary State Medical Association, Atlanta, Georgia.*

DEAR DOCTOR: In the light of the history, work and composition of the American Medical Association it is surprising, and seems almost incredible, that any one old enough, or considered intelligent enough, to practice medicine should charge it with having either the power or the purpose of swallowing up the State societies, or in any way interfering with their rights and privileges.

The Association is and has always been a purely voluntary organization, made up of such State and local organizations as like its methods and voluntarily join in its work for the improvement, elevation and protection of the profession.

Finding that Alabama and a few other States with almost universal county societies had been able to secure and enforce medical and health regulations, and in other ways better promote the interests of their physicians and people, and believing that many advantages would come from practical uniformity in methods over the entire country, the Association formulated a plan of organization for States and counties which has been unanimously adopted and put in operation in nearly every State in the Union. It would like to see the plan adopted in Georgia because it would be of inestimable benefit to the profession and people of the State, but it certainly has no selfish purpose to serve in the matter, and would only be indirectly benefited as your profession grew in power, influence and usefulness.

Under the old plan or the new one Georgia can affiliate with and send delegates to the Association just so long as it desires to do so and any individual can withdraw from the Association any day he sees proper to do so. It is glad to have you in affiliation with it, and would gladly welcome every reputable physician there or elsewhere into its membership, but it does not want any of them for a day longer than they want to stay. It would be unworthy to continue in existence if it did not stimulate advances in every department of knowledge and methods, and suggest and shape policies to these ends, but it has neither the desire or authority to compel any organization or individual to retain connection with it for a day longer than they find it to their pleasure or interest to retain the connection.

As the legislative body of the Association, which voices and directs its policies, is made up entirely of delegates from the State organizations, who in turn are elected by the county representatives, all, as nearly as possible, in accordance with our system of civil government, why should it want to swallow up or injure any of its component bodies? As no such thing has ever been attempted or even suggested by it or any one authorized to speak for it, and as no desire or power exists for doing so, the origin of such talk is hard to understand. Of course there are a few men in every State who were born in the objective case, and who oppose anything which did not originate with them, and while some of these in one or two States have been very noisy they have had little influence except where they were unknown. It must have started with them.

It may be proper for me to say in conclusion that I am an old-fashioned States-rights Democrat, that all of my people who were old enough fought in that cause whose survivors draw no pensions, and that every other member of my committee was as anxious as I was to safeguard in the plan every principle of local self-government, and every individual and State-right privilege, and that I feel sure this is done in the draft of the constitution and by-laws which you have under consideration.

Very respectfully,

J. N. McCORMACK.

---

*Dr. Louis H. Jones, Secretary Georgia State Medical Association, Atlanta, Ga.*

DEAR DOCTOR JONES: I take pleasure in answering your inquiry in regard to the relationship between this Association and the State societies.

This relationship has always been purely voluntary, and, in the very nature of such organizations, must always remain so. This is as true under the new plan as it was under the old one; no one wants to change it, and it could not be done if one did.

The accusation that the Association is urging the new plan through selfish purposes is so preposterous as to be wholly unaccountable. It can have no possible interest in it except in its desire and duty to unify and promote the growth and power of the profession of the whole country.

Since the American Medical Association is made up entirely of delegates from the State societies, whatever the majority of the delegates decide to do they can do. Hence, when our critics say that the Association is doing this, that or the other thing, they practically say that the State societies are doing these things. In other words, this Association is, and always will be, exactly what the State societies make it.

The new plan of organization is proving of inestimable advantage to all of the States which have had it under trial long enough to test its value, and we are only at the beginning of the good work. Of course it will be adopted in Georgia soon, even if it is not now, but the benefits to your profession would be so great that it would be a matter of sincere regret to all of us if misunderstandings or factious opposition should postpone action or mar the harmony of effectiveness of the work.

Let me emphasize that if the Medical Association of Georgia adopts the proposed constitution and by-laws, it will not in any way change its relation to the American Medical Association. Its members can join the A. M. A., or not, as they see fit, as at present. If they want to subscribe for The Journal, they can do so. The State Association will continue to be, as it is now, a separate and distinct body, subject only to such rules and regulations as it makes. It is my opinion that should your State Association decide not to adopt the proposed constitution and by-laws, its present relation to the A. M. A. will be unchanged, and the A. M. A. will recognize its delegates as in the past, it not being the wish of the A. M. A. to force any State society to act otherwise than as it sees fit.

Wishing you a pleasant and profitable meeting, and expressing the hope that great prosperity may come to your profession through the wisdom of its action, I am,

Very truly yours,

George H. Simmons, General Secretary.

